# Barriers to the early initiation of Insulin therapy among diabetic patients coming to diabetic clinics of tertiary care hospitals

**DOI:** 10.12669/pjms.35.1.237

**Published:** 2019

**Authors:** Iqra Arshad, Sara Mohsin, Sana Iftikhar, Tahseen Kazmi, Luqman F. Nagi

**Affiliations:** 1 Dr. Iqra Arshad, MBBS. Post Graduate Trainee Internal Medicine Services Hospital, Lahore, Pakistan; 2 Dr. Sara Mohsin, MBBS. PG Trainee Anesthesia, Mayo Hospital, Lahore, Pakistan; 3 Dr. Sana Iftikhar, MBBS. Assistant Professor, Department of Community Medicine, Shalamar Medical & Dental College, University of Health Sciences, Lahore, Pakistan; 4 Dr. Tahseen Kazmi, MPH, FCPS. Professor and Head of Department, Department of Community Medicine, Shalamar Medical & Dental College, University of Health Sciences, Lahore, Pakistan; 5 Luqman F. Nagi, MBBS, MPH. Assistant Professor, Department of Community Medicine, Shalamar Medical & Dental College, University of Health Sciences, Lahore, Pakistan

**Keywords:** Barriers, Insulin Therapy

## Abstract

**Background and Objective:**

Initiation of Insulin therapy during earlier stages has proved to significantly improve health outcomes among diabetics in comparison to oral medications. Not only patients but physicians are also often resistant to early initiation of insulin therapy. The objective was to assess misconceptions and barriers to early initiation of insulin therapy among diabetic patients coming to a diabetic clinic.

**Methods:**

This cross sectional study was conducted on 300 patients selected by convenience sampling arriving in Diabetes Outdoor Clinics of Mayo and Services Hospitals, Lahore during August 2017 to May 2018. The data was entered and analyzed by using SPSS version 17.

**Results:**

Out of 300 patients included in study, 39% (n= 117) were males and 61% (n=183) were females. The mean age of the participants at presentation was 48.46±13.15 years with a range of 13 to 80 years. Study participants considered it embarrassing to inject insulin in public place (p-value 0.01). The fear associated with lifelong commitment to insulin therapy once it is started, was also found statistically significant (p-value 0.001)particularly in subjects who have long duration of DM (>5 years).

**Conclusion:**

Perceptions of diabetic patients about insulin therapy are still barriers to early initiation of therapy and tend to prevail in Pakistan and around the globe.

## INTRODUCTION

Diabetes mellitus is a global health problem affecting both developed and resource-limited countries.^1^,^2^ Diabetes is a chronic, progressive illness that causes considerable morbidity related to macro- and micro vascular damage, resulting in psychosocial sequelae.^3^-^5^ Significant growing healthcare costs result due to overwhelming prevalence of Diabetes and associated disorders.^6^ It is estimated that approximately 9% of world population (over 415 million people) are suffering from Diabetes Mellitus worldwide.^6^ Diabetes contributed to five million deaths and 12% of total global adult health expenditure in 2015.^6^ Additionally another 642 million people are expected to suffer from diabetes by the year 2040.^6^ Pakistan had an estimated seven million diabetic population in 2015, and it is approximated to reach12 million in 2025 and 14 million by year 2040.^7^

The successful management of diabetes depends upon maintaining blood sugar levels within recommended target levels along with lifestyle modifications which include regular physical activities and dietary restrictions.^8^ Intensive glycemic control in patients with diabetes significantly reduces the risk of developing cardiovascular complications^9^ and also has a positive effect on long term management of both the macro and micro vascular outcomes of disease.^10^ Insulin should be initiated much earlier during the course of disease as this preventive strategy on individual level has proved to significantly improve the beta cell function among newly diagnosed diabetics in comparison to oral medications.^11^,^12^ Insulin delays the onset of diabetes related micro and macro vascular complications.^13^,^14^ Despite long term benefits of insulin therapy, unfortunately not only patients but physicians particularly primary healthcare providers are also often resistant to start or intensify insulin treatment, a phenomenon that has sometimes been referred to as psychological insulin resistance(PIR).^15^-^17^ Clinicians related barriers to initiating insulin therapy include alternative therapies, concern about hypoglycemia, monitoring of blood sugar levels and apprehension about weight gain with insulin.^18^,^19^ There is a widespread trend of misconceptions about insulin therapy in most parts of the world including Pakistan. The objective of this study was therefore to assess misconceptions among diabetic patients coming to a diabetic clinic about early initiation of insulin therapy.

## METHODS

A cross sectional study was conducted on patients with both Type-1 and Type-2 diabetes presenting to diabetic out-patient clinics at Mayo Hospital and Services hospital, Lahore to assess the barriers regarding early initiation of insulin therapy. A total of 300 patients participated in the survey conducted from August 2017 to May 2018. Informed consent was taken from all the participants during the survey and their identity and responses were kept confidential throughout the study. Structured questionnaires were administered by interviewers to all study participants in local language. In first half of questionnaire, demographic features were included. In second half, questions about diabetes diagnosis, duration and treatment modalities used to control diabetes were asked and in last half, questions regarding perceptions of patients about early initiation of insulin were assessed. The data was entered and analyzed by using SPSS version 17. Chi-square test was used for analysis of all variables and a P-value of < 0.05 was considered to have statistical significance.

## RESULTS

Out of 300 patients included in study, 39% (n=117) were males and 61% (n=183) were females. The mean age of the participants at presentation was 48.46 + 13.15 years with a range of 13 to 80 years. Among all, 29% (n=86) were illiterate, 28% (n=85) attained education up to secondary level (grade 8) and 43% (n=129) were matriculated and above. 89% (n=267) of study population had Type-2 and 11.0% (n=33) had Type-1 diabetes. Two types of subjects were studied, 82% (n=246) were those who had been prescribed insulin by specialist doctor and were using it and 18% (n=54) were those who had been prescribed insulin by a specialist doctor but denied to use it.

Out of 82% (n=246) of those using insulin, majority 41.7% (n=125) were using (70/30) (70% regular & 30% NPH) insulin, 24.3% (n=73) were using combinations of insulin i.e. Regular+ NPH, 12.3% (n=37) were using regular insulin only, 3.3% (n=10) were using NPH insulin only. When gender of study participants was cross tabulated with variables mentioned in Table-I, the participants considered it embarrassing to inject insulin in public place (p-value 0.01) and this perception was more common among females as compared to males. The perception about weight gain after starting insulin was different between males and females but was statistically insignificant (p-value 1.00). No statistical significance was found for variables such as side effects of insulin therapy, frequent shots of insulin, difficulty to self inject, interference with social life and belief that insulin can affect health negatively when cross tabulated with gender (Table-I).

**Table-I:** Gender of study participants and their perceptions about insulin use (n=300).

Perception of respondents	Male		Female		P-value

	Yes	No	Yes	No	
Side effects of insulin therapy	46	71	84	99	0.28
Dietary restrictions	96	21	133	50	0.07
Do you exercise daily?	59	58	71	112	0.05
Insulin more expensive than oral medications	97	20	143	40	0.37
Affordability of Insulin	69	48	127	56	.08
Challenging to take daily	58	59	94	89	0.81
Embarrassing to inject insulin in public place	54	63	114	69	0.01
Is it difficult to self-inject?	40	77	72	111	0.39
Have you noticed any weight change since starting insulin?	46	71	71	112	1.00
Frequent shots of insulin a problem	57	60	99	84	0.40
Fear of lifelong commitment to insulin once started.	89	28	144	39	0.59
Interference with social life.	41	76	72	111	0.46
Satisfaction of blood sugar control with insulin.	77	40	102	81	0.12
Improper counseling by doctor before starting insulin	22	95	39	144	0.66
Belief that insulin has negative effects on health.	16	101	35	148	0.27
Recommend insulin to others	78	39	118	65	0.86

Association of variables such as embarrassment of injecting insulin in public (p-value 0.05), insulin being more expensive than oral medications, affordability of insulin, and fear of lifelong commitment to insulin after starting were found to be statistically significant in patients with longer duration of diabetes of > 5years (Table-II). People with longer duration of diabetes prefer to recommend insulin to others having statistical significance of (p-value 0.000) because of potential benefits of good glycemic control. Perceptions of diabetic patients according to their educational level showed statistical significance with variables like side effects of insulin, difficulty to self-inject frequency of shots, fear of lifelong commitment to treatment and interference with social life (Table-III).

**Table-II:** Duration of diabetes mellitus and perceptions about insulin use (n=300).

Perception of respondents	Duration of Diabetes				P-value

	≤5 years		> 5 years		

	Yes	No	Yes	No	
Do you follow strict diet?	111	37	118	34	0.68
Do you exercise daily?	72	76	58	94	0.08
Side Effects of insulin	67	81	63	89	0.56
Affordability Of insulin	72	76	124	28	0.000
Is insulin more expensive than oral medications?	102	46	138	14	0.000
Embarrassing to inject insulin in public place	92	56	76	76	.05
Challenging to take daily	73	75	79	73	0.72
Is it difficult to self-inject insulin?	58	90	54	98	0.5
Have you noticed change in weight since starting insulin?	47	101	70	82	0.01
Fear of lifelong commitment to insulin.	102	46	131	21	0.001
Frequent shots a problem	74	74	82	70	0.56
interference of insulin therapy with social life	56	92	57	95	1.00
Belief that insulin has negative effects on health	22	126	29	123	0.35
Satisfaction of blood sugar control with insulin	77	71	102	50	0.02
Recommendation to others	78	70	118	34	0.000
Improper counseling by doctor prior to start insulin	26	122	35	117	0.25

**Table-III:** Education level of diabetic patients and their perceptions about insulin use (n=300).

Perception of respondents	Education level of Diabetes						P-value

	Illiterate		Up-to Grade 8		Matriculation or Higher		

	Yes	No	Yes	No	Yes	No	
Do you follow strict diet	65	21	62	23	102	27	0.57
Do you exercise daily?	31	55	37	48	62	67	0.21
Side effects of insulin	52	34	32	53	46	83	0.001
Affordability of Insulin	66	20	55	30	75	54	0.01
Insulin more expensive than oral medications	72	14	66	19	102	27	0.57
Challenging to take daily	52	34	40	45	60	69	0.09
Difficult to self- inject	54	32	26	59	32	97	.000
Embarrassing to inject insulin in public place	56	30	48	37	65	64	.13
Fear of lifelong Commitment to insulin	77	9	61	24	96	33	.02
frequent shots of insulin	55	31	45	40	56	73	.01
Have noticed changes in weight since starting insulin?	34	52	35	50	48	81	0.83
Interference of insulin therapy with social life	49	37	29	56	35	94	0.000
Belief that insulin has negative effects on health	23	63	14	71	14	115	0.01
Satisfaction of Blood sugar control with insulin	46	40	54	31	79	50	0.001
Recommendation of insulin s to other	54	32	59	26	83	46	0.7
Improper counseling by doctor about insulin	26	60	21	64	14	115	0.001

**Fig.1:**
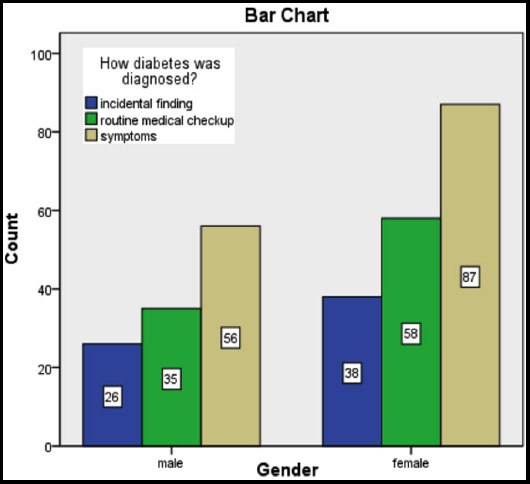


## DISCUSSION

In developing world, insulin therapy is not started earlier enough to achieve target glycemic goals to prevent complications of diabetes. It is delayed until it becomes an absolutely necessity or patient develops some complication.^22^ Myths and misconceptions about insulin use are one of the major reasons of such practice.^22^ These views are often reinforced by primary care physicians who themselves are resistant to early initiation of insulin therapy. Resistance to insulin therapy among type 2 diabetics not taking insulin (n=2061) and primary care providers i.e. nurses (n=1109) and physicians (n= 2,681) was studied in Cross National Diabetes attitudes, wishes and Needs (DAWN) study. In DAWN study, patients attitude towards insulin were studied through two variables i.e. perceived efficacy of insulin and self blame for needing insulin. In our study although we did not discuss physician’s barriers in initiating insulin but we assessed patient’s perception about efficacy of insulin through their opinion about side effects of insulin, satisfaction of glycemic control with insulin and recommendation to others and self blame through concerns that insulin means inability to control diabetes adequately resulting in lifelong commitment to insulin. We found that people having longer duration of disease, poor glycemic control and more advanced disease were satisfied about their glycemic control with insulin and willing to recommend insulin to others and were more concerned about lifelong commitment to treatment once started all variables were statistically significance (p value < 0.05) (Table 2). These findings are consistent with DAWN study results in which belief in insulin efficacy was stronger among patients who were in more negative situations i.e. more distressed about disease, worse control and more complications. Diabetes-related distress had the strongest association with insulin self blame (as it did with perceived benefits of Insulin therapy), with greater self-blame associated with more distress 20.

The results of a study entitled as Epidemiology and Diabetes Interventions and Complications (EDIC) study (2003) suggest that earlier the intensive therapy begins, the longer it is maintained and betters the chances of reducing the complications of diabetes.^21^

When assessed, the responses of participants for different possible barriers to insulin use (Table-I), it was found that injecting insulin in public place (p-value 0.001). Farsaei et al. in 2014 had discussed embarrassment about insulin injections (p-value <0.01) and concerns of weight gain with insulin (p-value 0.04) among diabetic patients both of Type-1 and Type-2 (n=508) as statistically significant in their study.^22^ These findings correspond well with our study (Table II). Bermeo Cabrera et al in their cross sectional study discussed behaviours and barriers in adherence to insulin therapy among type 2 diabetics in Mexico city and found that injecting insulin in public is embarrassing (p value 0.033), side effects of insulin injections i.e. pain and bruising (p value 0.002) and lack of economic resources to afford insulin (p value <0.0001) are potential reasons to omit insulin resulting in poor compliance. Also found the belief that insulin has negative effects on social and daily routine activities (p value 0.33) and insulin causes health problems e.g. blindness and kidney disease (p value 0.12) as statistically insignificant^23^. These results are comparable to our study findings.

Perceptions of patients about insulin therapy i.e. side effects of insulin therapy, problems with frequent insulin shots, difficulties in self administration, interference of insulin with social life and fear that insulin is treatment failure and once started has to be taken for whole life (Table-III) were assessed and found to be statistically significant (p<0.05) in our study. In our study, 18% (n=54) were totally unwilling to start insulin, 38% (n=113) thought that insulin interferes and restricts their social life and 78% (n=233) have perception that insulin is the last resort to cure diabetes and once started has to be taken for whole life. Polonski et al. (2005) discussed in their study on Type-2 diabetics (n=1,267) that 28.2% (n=357) patients were unwilling to start insulin, 44.8% (n=567) thought that insulin restricts life and 38% (481) believe that insulin is associated with more advanced stage disease.^17^

Gutierrez et al. (2015) had conducted a systematic review of studies related to insulin therapy misconceptions among Hispanics populations published during the last 22 years (1992-2014). His main findings were that negative attitudes about insulin therapy was associated with more severe disease, expense of insulin, administration difficulties and a overall negative impact of insulin on life-style among Latinos / Hispanics diabetics. General attitudes of people towards insulin therapy are comparable in both studies.^24^ Results of an study conducted in India on patients with diabetes (n=52) who had failed to control their disease with oral anti-diabetic agents, 69% (n=36) declined to start insulin in comparison to 18% (n=54) in our study and 50% (n=26) believed that insulin damaged other organs and had negative effects on overall health compared to 23% (n=69) in our study.^25^

The global attitudes of patients and physicians in insulin therapy (GAPP) study by Peyrot et al. in 2012 had identified factors responsible for non-compliance among diabetic patients globally. Authors found that non-adherence to insulin were quite common and significantly higher among those who believed that insulin therapy impacts lifestyle. Furthermore non-adherence was also found significantly higher among those who considered insulin injections difficult to administer and insulin regimen less flexible to manage.^26^

### Limitations of the study

It includes convenient sampling and small sample size. Due to small sample size and data collection from only two diabetic centers, the perceptions of diabetic patients about early initiation of insulin therapy cannot be generalized to all diabetic patients.

## CONCLUSION

Patient’s misconceptions about insulin therapy are major barriers to early initiation of therapy. These perceptions prevail more in areas where patients belong to poor socioeconomic background and have low education levels resulting in improper education about the long term benefits of insulin therapy. In order to initiate insulin therapy earlier in disease process and to avoid serious complications resulting from poor glycemic control of diabetes, health education of both patients and primary healthcare physicians is warranted.

### Author`s Contribution

**IA** did data collection and manuscript writing.

**SM** did data collection.

**SI, LFN and TK** did the design, statistical analysis, editing, review and final approval of manuscript.
